# Psychological and Social Work Factors as Predictors of Mental Distress: A Prospective Study

**DOI:** 10.1371/journal.pone.0102514

**Published:** 2014-07-21

**Authors:** Live Bakke Finne, Jan Olav Christensen, Stein Knardahl

**Affiliations:** 1 Department of Work Psychology and Physiology, National Institute of Occupational Health, Oslo, Norway; 2 Department of Psychology, Norwegian University of Science and Technology, Trondheim, Norway; 3 Department of Psychology, University of Oslo, Oslo, Norway; University of Regensburg, Germany

## Abstract

Studies exploring psychological and social work factors in relation to mental health problems (anxiety and depression) have mainly focused on a limited set of exposures. The current study investigated prospectively a *broad set* of *specific* psychological and social work factors as predictors of potentially clinically relevant mental distress (anxiety and depression), i.e. “caseness” level of distress. Employees were recruited from 48 Norwegian organizations, representing a wide variety of job types. A total of 3644 employees responded at both baseline and at follow-up two years later. Respondents were distributed across 832 departments within the 48 organizations. Nineteen work factors were measured. Two prospective designs were tested: (i) with baseline predictors and (ii) with average exposure over time ([T1+T2]/2) as predictors. Random intercept logistic regressions were conducted to account for clustering of the data. Baseline “cases” were excluded (n = 432). Age, sex, skill level, and mental distress as a continuous variable at T1 were adjusted for. Fourteen of 19 factors showed some prospective association with mental distress. The most consistent risk factor was *role conflict* (highest odds ratio [OR] 2.08, 99% confidence interval [CI]: 1.45–3.00). The most consistent protective factors were *support from immediate superior* (lowest OR 0.56, 99% CI: 0.43–0.72), *fair leadership* (lowest OR 0.52, 99% CI: 0.40–0.68), and *positive challenge* (lowest OR 0.60, 99% CI: 0.41–0.86). The present study demonstrated that a broad set of psychological and social work factors predicted mental distress of potential clinical relevance. Some of the most consistent predictors were different from those traditionally studied. This highlights the importance of expanding the range of factors beyond commonly studied concepts like the demand-control model and the effort-reward imbalance model.

## Introduction

Mental health problems represent an increasingly important public health challenge. The World Health Organization has pointed to depression as a leading cause of the disease burden (measured in disability-adjusted life years) in middle- and high income countries [1].

The workplace presents individuals with a variety of challenges from work tasks and social interactions. Work may also provide opportunities for positive achievement, fulfilment, and friendship. For many the job is a crucial source of feedback and may be a central component of personal identity. Thus, working conditions may represent a particularly salient influence on emotions, self-esteem, and identity. Although employment is commonly assumed to promote health, the net effect on mental health depends on the psychosocial *quality* of work [2]. Knowledge of specific aspects of work that influence mental health should provide a practical basis for organizational improvements by directing the focus of surveys and interventions to improve employee health. Therefore, the present study sought to identify *specific* occupational psychological and social factors that predict subsequent mental distress (anxiety and depression) of *potential clinical relevance*. Unlike much previous research, the current study included a wide range of specific exposures covering both task-, individual-, and social- and organizational level factors, to compare the relevance of numerous work factors to mental distress.

Prospective studies have shown that high demands, low control, low social support, effort-reward imbalance, organizational injustice, job insecurity, undesirable work events, and bullying contribute to common mental disorders (anxiety and depression) (systematic reviews: [3]–[9]). However, the research has been dominated by the demand-control (DC) (e.g. [10]) and the effort-reward imbalance (ERI; e.g. [11]) models. These models have been pivotal in generating the present state of knowledge, but a consequence of their dominance is a low level of evidence for many other work factors.

The constructs demands and control of the demand-control model are well-defined broad dimensions [12]. However, common operationalisations of the constructs have grouped together factors that may produce very different effects. Almost all studies have measured demands and control by the Job Content Questionnaire (JCQ) [10]. This instrument measures “demands” by questions pertaining to time pressure, amount of work, and role conflicts. There are several types of demands (e.g. quantitative, qualitative, positive challenges, etc) and role conflicts may produce different health effects than demands (e.g. [13], [14]). The job control dimension (“decision latitude”) of the JCQ includes both “skill discretion” (variety of work and opportunity to use skills) and “decision authority” (control over decisions that influence work) which may also affect health differentially (see e.g. [15]). High levels of skill discretion may imply more responsibility and may be conceptually related to demands and positive challenges. Similarly, both factors of the ERI instrument are general and rather unspecific. Effort results from both job demands and the individual trait-like factor commitment, while reward includes both feedback and job security [11]. Furthermore, some measures of relational justice of the “organizational justice” concept include both feedback and truthfulness of one's superior [16]. The focus on a few broad dimensions may imply that many previous studies have not provided knowledge that is sufficiently specific to direct effective workplace interventions. A recent systematic review reported that intervention practices for depression in the workplace have not yet been able to demonstrate robust positive results [17]. After numerous studies devoted to testing general models it may be timely to investigate effects of specific exposures and to extend the scope to a wider range of exposures [3], [6], [7].

A recent attempt to broaden the scope of psychological work exposures considered health-relevant has been the formulation of the *Job Demands Resources model* (JD-R) [18], [19]. This model has gained widespread popularity over the past decade. The authors of the model place emphasis on testing a general theory of psychological work exposure at a high level of abstraction. Originally a model to explain burnout, this model has been proposed as an aid to theoretical development and an alternative to a so-called “laundry list”-approach in which different exposures are studied simultaneously without a comprehensive theory [18]. However, while the JD-R approach focuses on assessing a theoretical model, the interest of the current study was in uncovering specific precursors of the studied health affliction. Models or theories are necessary to generate and test general “laws” or relationships that promote general understanding. However, knowledge of specific exposures may be applicable to interventions even when not part of a model. In fact, many studies investigating demands and control test the dimensions separately. For instance, Bosma and coworkers [20] reported that control, but not demands and support, was associated with coronary heart disease. Investigating specific factors is not inferior to testing a model of a combination of factors unless the predictions of the model are the primary subject of investigation.

The central assumption of the JD-R model is that work factors can be classified into two general categories: job demands and job resources. *Job demands* refer to any aspect of work that requires sustained effort and is thereby “associated with certain physiological and/or psychological costs” ([18], p. 312). *Job resources* refer any aspects of the job that are “either/or: functional in achieving work goals, reduce job demands and the associated physiological and psychological costs, or stimulate personal growth, learning, and development” ([18], p. 312). Hence, exposure is defined by its consequences and thus the predictions of the model seem circular (“demands” are anything that has adverse health impacts). The authors of the model have conceded that some job demands may be “good stressors” or “challenge stressors” and others “hindrance stressors” [21], but this is not readily integrated into the model and it is difficult to see how such demands would fit into the above cited definition. Studies have shown various “demands” to be related to emotional exhaustion and “resources” to engagement, but mostly in small cross-sectional samples (for listing of studies see e.g. [18], [22]). The most studied outcome in JD-R research, *burnout* (consists of emotional exhaustion, depersonalization, and personal accomplishment) differs from *mental distress* (symptoms of anxiety and depression) (see e.g. [23]–[29]). Furthermore, to our knowledge no previous studies have included as broad a set of factors as the current study.

Mental health is multidimensional and many of the constructs describing dimensions are overlapping. Mental health problems and negative mental states are commonly labelled mental distress [26], [27] or negative affect [30]. Both constructs are loosely defined and usually incorporate depressive emotions, anxiety, and other negative emotional states. Depression is often defined by sadness, loss of initiative, and self-blame [23], [24]. Emotional exhaustion, one of three components of the burnout syndrome [31], is related to depressive emotions and loss of initiative [32]. Feeling tired or fatigued is common and may be related to loss of initiative.

Much previous research has been confined to one type of occupation or one single workplace. Also, many prospective studies have investigated effects of exposure measured at one time point on subsequent health. As the time required before an exposure becomes harmful and potentially invokes mental distress is unknown [33] and exposure may fluctuate over time, systematic reviews have emphasised the need for investigating duration of exposure [4]–[7]. The risk of ill health may be higher when challenging conditions are an integral part of the general situation at work than when challenges are encountered as single events or periods. Thus, if employees experience alleviation of exposure during the follow-up period of a study adverse effects may not develop. Also, if health effects did in fact occur, health may be restored at the end of the study. Thus, designating exposure based on one time point only may constitute misclassification. Measuring exposure twice should yield more “reliable” representations of the overall working conditions over the time period in question by attenuating the influence of occasion-specific factors and random fluctuations.

The present study included some specific work factors that to our knowledge have not previously been investigated as predictors of mental distress (anxiety and depression) in prospective studies: *predictability during the next month, predictability during the next two years, human resource primacy*, and *empowering leadership*. We also examined factors that have received little attention (*control over work intensity, role conflict, role clarity, rumors of change, fair leadership, social climate, commitment to organization*, and *observed bullying*) as well as established risk factors (*quantitative demands, decision demands, decision control, positive challenge, supportive leadership, procedural justice*, and *experienced bullying*). The theoretical background of the psychological and social work factors included in the present study can be found in Lindström et al. [34]. The outcome was mental distress of potential clinical significance (i.e. “caseness”) (see [28], [29]). Important features of the present study were inclusion of several types of jobs and the full-panel design. The repeated measurement of exposures made it possible to test several designs in order to elucidate which factors show the most robust associations with distress. Furthermore, the full-panel design allowed the estimation of exposure over time.

## Methods

### Ethics statement

This study has been approved by the Regional Committees for Medical and Health Research Ethics (REK) in Norway, has permission from the Data Inspectorate of Norway and was conducted in accordance with the World Medical Association Declaration of Helsinki. All study participants provided their informed consent. When accessing the web-based questionnaire by a personal login code, informed consent had to be confirmed before responding to the questionnaire. This consent procedure was approved by the Data Inspectorate of Norway and REK. Data were analyzed anonymously.

### Design

The study was a prospective two-wave full-panel design. Average follow-up period was 24 months (range 17–36). This study is part of a comprehensive project assessing a wide range of work factors and outcomes, and a two-year time-lag was considered the best to capture the various processes under study. Furthermore, this time-lag was what worked best for the participating companies. A time-lag of at least two years (compared with a four-year time-lag) has been shown adequate to demonstrate a relationship between stressors at work, irritation, and depressive symptoms [35]. However, the paucity of knowledge of pathogenic mechanisms precludes the design of an optimal exposure-outcome measurement interval.

The prospective relation of psychological/social work factors with mental distress was tested by two statistical designs: (i) modeling incidence of mental distress (at T2) as a function of exposures at baseline and (ii) modeling mental distress (at T2) as a function of average exposure over time ([T1+T2]/2).

### Subjects

Subjects were recruited from 48 Norwegian organizations (31 private and 17 public) that were contacted and offered participation. Hence, a *convenience sampling technique* was applied. The invited subjects were distributed across 1158 departments within the organizations. Average number of subjects in each department was 10, ranging from 1 to 159 individuals. In return for participating, organizations received written reports and oral presentations of results of the work environment survey. Baseline data were collected from November 2004 until June 2009, and follow-up data from September 2006 until June 2011. The organizations included municipalities, an insurance company, public organizations, health institutions, and educational institutions, among others, representing a wide variety of job types.

Employees and management were informed of the project at the organizational level. The organizations supplied lists of names, addresses, sex, age, personal identification numbers, departmental affiliation, and classification of the occupations of all their employees. Subsequently, all employees were mailed letters with information of the purpose of the study and confidentiality, and either a personal access code to the web-based questionnaire or a paper version of the questionnaire. For details about the data collection procedure, see Christensen and Knardahl [13].

Occupation was classified according to the standard classification of occupations (STYRK), developed by Statistics Norway (www.ssb.no) based on the International Standard Classification of Occupation (ISCO-88). One criterion for this classification is the technical and formal skills normally required for a certain occupation. Required skills do not have to be obtained by formal education, but should reflect the education level equivalent to a certain skill level (Table 1). In the present study the variable *skill level* was included as a proxy for education.

**Table 1:** Baseline characteristics of respondents to the first survey^a^, respondents to the first and second surveys^b^, and drop-outs from the first to the second survey.

	Invited to the first survey (N = 12 603)					Invited to the first and second surveys (N = 9304)						
	Respondents to the first survey (N = 6506)		Non-response analyses^c^			Respondents to the first and second surveys (N = 3644)		Drop-outs from the first to the second survey (N = 1760)		Attrition analyses^c^		
	N	%	N	OR	95% CI	N	%	N	%	N	OR	95% CI
**Sex**	.	.	.	.	.	.	.	.	.	.	.	.
Male	2547	39.1	4682	1.00	ref	1475	40.5	646	36.7	2121	1.00	Ref
Female	3956	60.8	7389	0.97	0.90–1.04	2169	59.5	1114	63.3	3283	0.85	0.76–0.96*
Missing data	3	0.1	.	.	.	0	0	0	0	.	.	.
**Age**	.	.	.	.	.	.	.	.	.	.	.	.
<30	596	9.2	1279	1.00	ref	237	6.5	158	9.0	395	1.00	ref
30–39	1716	26.4	3073	1.45	1.27–1.65*	913	25.1	458	26.0	1371	1.33	1.06–1.67*
40–49	1982	30.5	3500	1.50	1.32–1.70*	1196	32.8	534	30.3	1730	1.49	1.19–1.87*
50–59	1679	25.8	3067	1.39	1.22–1.58*	1042	28.6	483	27.4	1525	1.44	1.14–1.81*
>59	527	8.1	1149	0.97	0.83–1.14	256	7.0	127	7.2	383	1.34	1.00–1.80
Missing data	6	0.1	.	.	.	0	0	0	0	.	.	.
**Classification of occupation**	.	.	.	.	.	.	.	.	.	.	.	.
Legislators, senior officials, and managers	608	9.3	.	.	.	396	10.9	119	6.8	.	.	.
Professionals	1776	27.3	.	.	.	1042	28.6	402	22.8	.	.	.
Technicians and associate professionals	2191	33.7	.	.	.	1199	32.9	642	36.5	.	.	.
Clerks	536	8.2	.	.	.	283	7.8	169	9.6	.	.	.
Service workers and shop and market sales workers	1088	16.7	.	.	.	571	15.7	315	17.9	.	.	.
Skilled agricultural and fishery workers	2	0.0	.	.	.	1	0.0	1	0.1	.	.	.
Craft and related trades workers	83	1.3	.	.	.	42	1.2	23	1.3	.	.	.
Plant and machine operators and assemblers	10	0.2	.	.	.	1	0.0	7	0.4	.	.	.
Elementary occupations	86	1.3	.	.	.	46	1.3	30	1.7	.	.	.
Armed forces and unspecified	32	0.5	.	.	.	21	0.6	11	0.6	.	.	.
Missing data	94	1.4	.	.	.	42	1.2	41	2.3	.	.	.
**Skill level**	.	.	.	.	.	.	.	.	.	.	.	.
Competence equivalent to minimum 4 years of higher education (>16 years)	1776	27.3	.	.	.	1042	28.6	402	22.8	1444	1.00	ref
Competence equivalent to 1–3 years of higher education (13–15 years)	2191	33.7	.	.	.	1199	32.9	642	36.5	1841	0.72	0.62–0.84*
Competence equivalent to high school (10–12 years)	1719	26.4	.	.	.	898	24.6	515	29.3	1413	0.67	0.57–0.79*
Occupations that do not require high school (<10 years)	86	1.3	.	.	.	46	1.3	30	1.7	76	0.59	0.37–0.95*
Occupations with unspecified requirements for competence	640	9.8	.	.	.	417	11.4	130	7.4	547	1.24	0.99–1.55
Missing data	94	1.4	.	.	.	42	1.2	41	2.3	.	.	.
**Mental distress**	.	.	.	.	.	.	.	.	.	.	.	.
<1.85	5667	87.1	.	.	.	3212	88.1	1532	87.0	4744	1.00	ref
≥1.85	839	12.9	.	.	.	432	11.9	228	13.0	660	0.90	0.76–1.07
Missing data	0	0	.	.	.	0	0	0	0	.	.	.

a Respondents were defined as having completed the HSCL-10, minimum one predictor at the first survey, and having information on department affiliation.

b Respondents were defined as having completed the HSCL-10 at both the first and second surveys, minimum one predictor at the first survey, and having information on department affiliation at the first survey.

c Separate univariable logistic regression analyses to estimate non-response and attrition.

*p<.05.

The questionnaire gathered data about background information, work organization, psychological/social work factors, organizational change, attitudes to work, personality, coping strategies, physical activity, smoking, alcohol use, mental health, work ability, and health complaints. This study is based on parts of this information.

**Figure 1:**
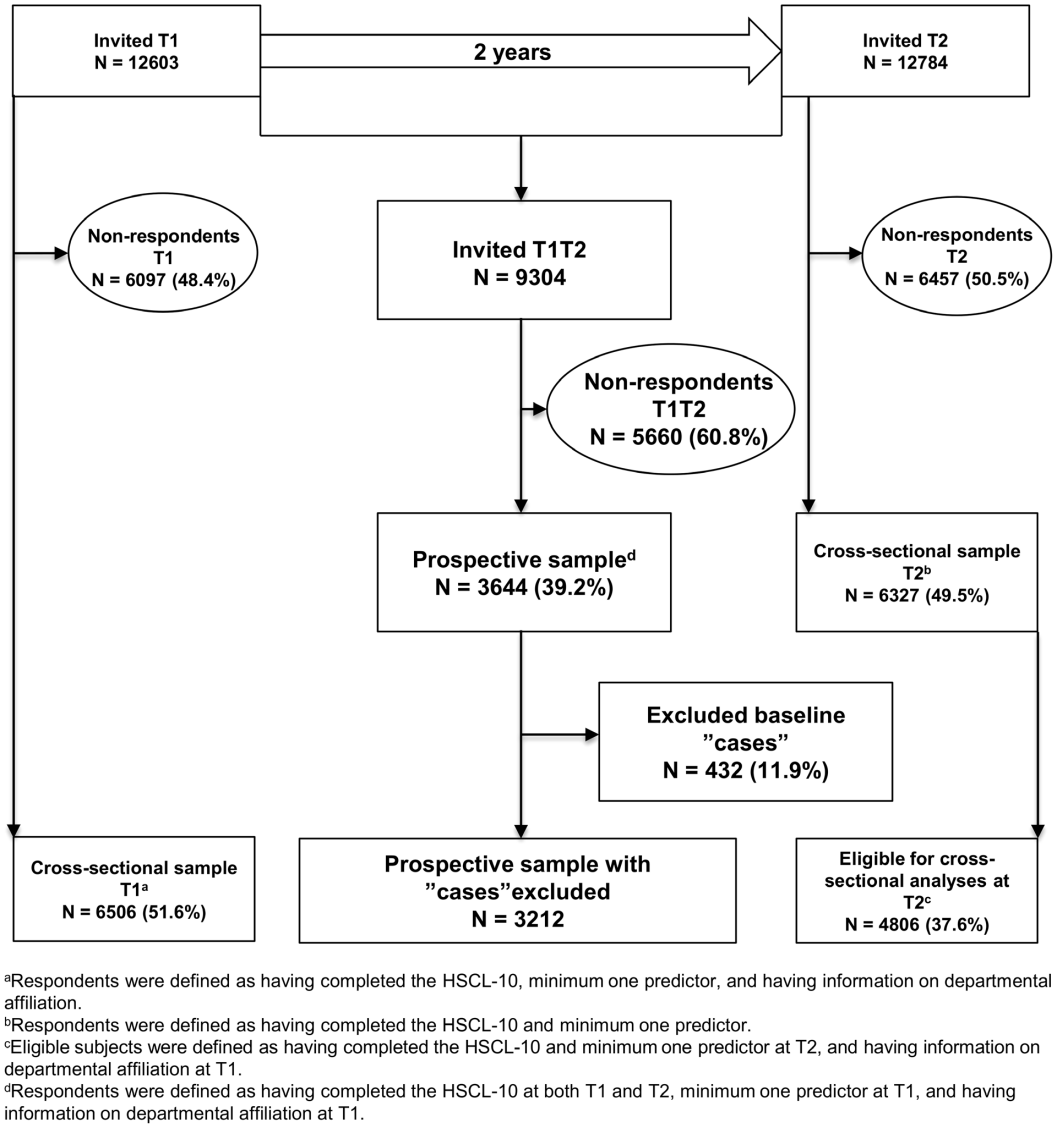
Study design and response rates.

At baseline, 12603 subjects were invited, of which 6506 (51.6%) were characterized as respondents (Figure 1). Response was defined as having completed minimum one psychological/social work factor, the outcome measure Hopkins Symptom Checklist (HSCL-10), and having information on departmental affiliation. Departmental affiliation was needed as this was used as the cluster variable to account for clustering of the data in the statistical analyses. The respondents were distributed across 993 departments within the 48 organizations. Average number of employees in each department was 7, ranging from 1 to 55 individuals. These subjects constituted the *cross-sectional* sample at T1. At follow-up, 12784 were invited. Of these, 6327 (49.5%) responded to minimum one work factor and the HSCL-10. However, as information on departmental affiliation only was available for those who were also invited at T1, only 4806 (37.6%) subjects were eligible for *cross-sectional analyses* at T2. These subjects were distributed across 934 departments within the 48 organizations with an average number of 5 (range 1–38) individuals in each department. Some employees left or entered companies during the follow-up period and were thus invited only once. Therefore, the cross-sectional samples only partially overlapped. There were 9304 employees who were invited at both time points. Of these, 3644 (39.2%) responded at both T1 and T2. The respondents were distributed across 832 departments within the 48 organizations with an average number of 4 (range 1–33) individuals in each department. Of the 3644 subjects, 432 reported mental distress at baseline and were thus excluded from prospective analyses. Hence, 3212 employees were eligible for prospective analyses (Figure 1).

### Measures

#### Outcome: mental distress

A Norwegian translation of the Hopkins Symptom Checklist-10 (HSCL-10) measured degree of mental distress (symptoms of anxiety and depression) *during the last week*. HSCL has shown adequate psychometric properties [27] and is a frequently used self-report instrument to assess mental distress in population surveys [26]. HSCL-10 is an abbreviated version of HSCL-25.The correlation between these instruments was 0.97 in a previous validation study [29]. While HSCL-25 distinguishes between depression and anxiety [26], it has been suggested that HSCL-10 is a one-dimensional measure of general mental distress [36]. Examples of items in HSCL-10 are “feeling tense or keyed up” and “feeling hopeless about the future”. Responses are given on a four-point scale: 1 = “Not at all”, 2 = “A little”, 3 = “Quite a bit”, and 4 = “Extremely”. Missing values were replaced with the individual mean, but responders with three or more missing items were excluded. This constituted 32 (0.5%) responders at T1 and 23 (0.5%) at T2, respectively. Cronbach's α for this scale was 0.87 at both T1 and T2.

To define “cases”, HSCL-10 was dichotomized at the 1.85 level. In a representative sample of the Norwegian population this cut-off level has been shown to correspond to the conventional cut-off of 1.75 on HSCL-25 [29] which predicts mental disorder as assessed independently by clinical interview (e.g. [28]).

#### Psychological and social work factors

Psychological and social work factors were assessed by the General Nordic Questionnaire for Psychological and Social Factors at Work (QPS_Nordic_) [37]. QPS_Nordic_ has been thoroughly tested for validity and reliability and has shown good psychometric properties [37], [38].

The following fifteen scales were studied; *quantitative demands* (i.e. time pressure and amount of work), *decision demands* (i.e. demands for decision-making and attention), *decision control* (i.e. influence on decisions regarding work tasks, choice of coworkers, and contacts with clients), *control over work intensity* (i.e. influence on time, pace, and breaks), *role conflict* (i.e. conflicts between demands and resources, conflicting requests), *role clarity* (i.e. clarity of goals and objectives at work), *support from immediate superior* (i.e. instrumental and emotional support, and appreciation), *empowering leadership* (i.e. encouragement for participation in important decisions and expressing differing opinions, development of skills), *fair leadership* (i.e. distribute work fairly and treat workers fairly and equally), *predictability during the next month* (i.e. predictability of tasks, coworkers, and superiors), *predictability during the next two years* (i.e. predictability of job security and learning demands), *commitment to organization* (i.e. positive feelings and attitudes towards the workplace), *social climate* (i.e. whether the social climate is encouraging/supportive, distrustful/suspicious, relaxed/comfortable), *positive challenge at work* (i.e. usefulness of skills and knowledge, meaningfulness of work, and if work is challenging in a positive way), and *human resource primacy* (i.e. organizational practices pertaining to rewarding workers for well-done jobs, taking good care of workers, the interest of management in the health and well-being of workers). The scales varied from three to five items. Cronbach's alphas ranged from 0.64 to 0.91 at baseline and from 0.64 to 0.92 at follow-up.

Response scale was: “1 = very seldom or never”, “2 = somewhat seldom”, “3 = sometimes”, “4 = somewhat often”, and “5 = very often or always”. Exceptions were *commitment to organization* with the response alternatives: “1 = disagree totally”, “2 = disagree to some extent”, “3 = indifferent”, “4 = agree to some extent”, and “5 = agree totally” and *predictability during the next two years, human resource primacy*, and *social climate*: “1 = very little or not at all”, “2 = rather little”, “3 = somewhat”, “4 = rather much”, and “5 = very much”.

Three *single items* from QPS_Nordic_ were also included. “Are there rumors concerning changes at your workplace?” with the response scale “1 = very seldom or never” to “5 = very often or always”. *Observed bullying* was measured by: “Have you noticed anyone being subjected to harassment or bullying at your workplace during the last six months?” and *experienced bullying* by the “subjective” method (see [39]): “Have you been subjected to bullying or harassment at the workplace during the last six months?”. The response categories for both items were “yes” and “no”. Respondents were presented with a definition of bullying and harassment (for definition see [40]).

A single question measured *organizational procedural justice* [41] related to organizational change: “Procedures are designed to hear the concerns of all those affected by the decision” with the response alternatives “1 = strongly agree”, “2 = quite agree”, “3 = neutral”, “4 = quite disagree”, and “5 = strongly disagree”.

### Statistical analyses

Statistical analyses were conducted with SPSS Statistics, version 19.0 (IBM, Armonk, NY, USA), Mplus Version 6.11 [42], and R Version 3.0.2 [43].

The association of sex and age with *non-response* was estimated with univariable logistic regression analyses. All individuals invited at baseline were included in the analyses.

*Attrition bias* was tested with logistic regressions. For baseline responders, the odds of *also* responding at follow-up were computed. Predictors in univariable regressions were age, sex, skill level, mental distress (T1), and psychological/social factors. Statistically significant predictors were subsequently entered in a multivariable regression.

Univariable and multivariable random effects logistic regression analyses were conducted to estimate the relationship between work factors and mental “caseness”. The subjects were clustered in organizations and departments. Thus, lack of independence between observations may exist in the data. Standard regression modelling is based on the assumption of independent observations and applying such statistical tests for clustered data could generate inaccurate estimates. By employing random effects, clustering or potential lack of independence that may exist in the data is accounted for [44]. Departmental affiliation was used as the cluster variable. The 48 organizations were very different both in size and structure (ranging from 13 to 2317 employees) and in type (including municipalities, an insurance company, public organizations, health institutions, and educational institutions, among others). In many of the large organizations employees occupied a wide variety of job types *and* were geographically dispersed. Thus, employees seemed more likely to be influenced by a shared work environment within their department than at the organizational level. Therefore, departmental affiliation was treated as the cluster variable. Both random intercept (i.e. allowing the intercept to vary across departments) and random slope (i.e. allowing the regression coefficients to vary across departments) models were tested. The Bayesian information criterion (BIC) was employed to decide whether intercept only or intercept and slope models should be preferred. The model with the lowest BIC value is the better fitting model [45].

To establish associations, regressions were executed *cross-sectionally* at T1 and T2 prior to *prospective analyses*. As the cross-sectional samples contained both respondents who only responded at one time point *and* those who responded at both time points these analyses should provide some additional information on the reliability of the associations across samples. Cross-sectional analyses at T2 were conducted both with those who were also invited at T1 (and had information on departmental affiliation) as well as with *all* who were invited at T2 that responded to HSCL-10 and minimum one work factor. Results from these two samples were very similar. Here, only results from the analyses with responders who had information on departmental affiliation are reported. *Prospective analyses* estimated the effect of levels of exposure both at (A) baseline and (B) averaged over time ([T1+T2]/2). The baseline model estimated possible long-term *effects* while the average model estimated effects of long-term *exposure*.

Examining a broad set of factors necessitated multiple testing. To reduce the risk of type I error, 99% confidence intervals were employed. In addition, a Bonferroni-adjusted threshold of statistical significance was applied. This was estimated by dividing the overall significance level by the number of factors tested (i.e. 0.01/19 = 0.0005). Age, sex, and skill level were included in all multivariable analyses. To predict the incidence of potentially clinically relevant mental distress, individuals classified as “cases” at baseline (n = 432; Table 1) were excluded. Mental distress as a continuous variable at T1 was adjusted for.

As the objective of the current study was to explore a broad set of exposures to *identify* predictors of mental distress, each work factor was modeled separately both cross-sectionally and prospectively. Mutually adjusting for all other exposures in this comprehensive study would diminish statistical power and constitute overadjustment. This is particularly inappropriate if the included factors are causally related in other ways, for instance by mediating the effects of each other. Statistical procedures alone cannot distinguish between mediation and confounding [46]. Previous research identifying confounders is to our knowledge lacking, and “blindly” entering control variables into models may severely underestimate effects [4]. Testing how the work factors may interact in complex mechanisms influencing mental distress was beyond the scope of the current study. As several of the work factors included in this comprehensive study have not previously been investigated (see the introduction) it is necessary to identify and “map out” predictors of mental distress in advance of testing possible interactions in future studies.

## Results

### Baseline characteristics

Among respondents to the first survey (T1) the three largest occupational groups were *technicians and associate professionals* (N = 2191, 33.7%), *professionals* (N = 1776, 27.3%), and *service workers and shop and market sales workers* (N = 1088, 16.7%) (Table 1). The corresponding figures for responders at both baseline and follow-up were 32.9% (N = 1199), 28.6% (N = 1042), and 15.7% (N = 571).

Among T1 responders 12.9% (N = 839) met the criterion for mental “caseness” (≥1.85) (Table 1). The prevalence was 11.9% (N = 432) among responders to both T1 and T2. These prevalences are similar to the one observed in a representative sample of the Norwegian population [29]. The incidence of mental distress during the follow-up period was 6.8% (N = 219).

### Non-response and attrition analyses

The three middle age groups (30–39, 40–49, and 50–59) displayed statistically significantly increased odds of responding compared to the lowest age group (<30) (Table 1). Sex was not associated with responding.

For baseline responders, mental distress did not predict responding at follow-up. Being female lowered the odds of responding at follow-up. Age groups 30–39, 40–49, and 50–59 were associated with higher odds of responding compared to the youngest (<30). Employees in occupations requiring the equivalent of >16 years of education displayed higher odds of responding than the three middle groups (13–15, 10–12, and <10 years of education) (Table 1).

*Age, skill level*, and *role clarity* were statistically significant predictors of responding at follow-up in the multivariable attrition analysis (analysis not shown). Age group 50–59 (odds ratio [OR] 1.37, 95% confidence interval [CI]: 1.05–1.78) displayed higher odds of responding. The group of occupations that do not require high school (OR 0.50, 95% CI: 0.27–0.92) and the groups with requirements of competence equivalent of 10–12 years (OR 0.75, 95% CI: 0.61–0.92), and 13–15 years (OR 0.76, 95% CI: 0.63–0.91) of education exhibited lowered odds of responding. Higher scores on role clarity were associated with decreased odds of responding at follow-up (OR 0.81, 95% CI: 0.73–0.90).

### Cross-sectional analyses

Univariable random intercept logistic regressions with sex, age, and skill level as independent variables and the dichotomized HSCL-10 as outcome were conducted in the T1 sample (analyses not shown). Women showed increased odds for experiencing mental distress (OR 1.28, CI: 95% 1.09–1.49). Lowered odds of mental distress were observed for the “unspecified” category of the skill level classification (OR 0.52, CI: 95% 0.38–0.72). Age was not associated with mental distress (p>.05).

Univariable *and* multivariable random intercept regressions revealed statistically significant associations for all factors except *decision demands* with mental distress at T2 (analyses not shown). All factors except *decision demands* at T1 remained statistically significant after Bonferroni-correction in both univariable and multivariable analyses. Statistically significant ORs in multivariable regressions ranged from 0.40 (*social climate* at T2) to 4.93 (*experienced bullying* at T1).

For all cross-sectional models the BIC (Bayesian information criterion) values were smaller for models including a random intercept only compared to models including both random intercept and random slope (not shown). The difference was above 2 for all models, which constitutes positive evidence for random intercept only models to have best fit to the data [45]. For most models except *rumors of change* at T1 (both univariable and multivariable models) and *decision demands* (multivariable model), *human resource primacy* (univariable and multivariable models), and *rumors of change* at T2 (multivariable model), the difference in BIC was >10, which according to Raferty [45] is considered “very strong” evidence for a better fit to the data. Hence, the additional complexity of adding random slopes to the regression models was not considered justified.

### Prospective analyses: work factors as predictors of new “cases” of mental distress

#### Baseline exposure as predictor

Univariable random intercept regressions revealed that 13 of 19 factors predicted mental distress at follow-up (p<.01; analyses not shown). All factors except *commitment to organization*, *procedural injustice*, and *observed bullying* remained statistically significant after Bonferroni-correction. *Quantitative demands, decision demands, control over work intensity, predictability during the next month, predictability during the next two years*, and *rumors of change* were not predictors (p>.01). Adding random slopes to the models did not improve the fit. The BIC values were smaller for random intercept models with differences of >10 for all models (not shown).

Multivariable analyses showed that four work factors were statistically significant predictors. *Role conflict* was associated with increased odds of mental distress. *Support from immediate superior, fair leadership*, and *positive challenge* lowered the odds. Role conflict remained statistically significant after Bonferroni-correction (Table 2). Random intercept models showed the best fit to the data with between-model differences in BIC values of >10 for all models (not shown).

**Table 2:** Multivariable random intercept logistic regression models with psychological and social work factors at T1 as predictors of mental distress above the cut-off for "caseness" at T2^a^.

Exposure	N	OR	99% CI
Quantitative demands	3090	1.06	0.81–1.38
Decision demands	3033	0.75	0.55–1.04
Decision control	2969	0.75	0.56–1.02
Control over work intensity	3130	0.90	0.71–1.14
Role conflict	3137	1.53	1.15–2.05***
Role clarity	3144	0.84	0.65–1.08
Support from immediate superior	3107	0.76	0.60–0.95**
Empowering leadership	3136	0.87	0.71–1.07
Fair leadership	3108	0.75	0.59–0.96**
Predictability during the next month	3149	0.87	0.67–1.13
Predictability during the next two years	2901	0.95	0.78–1.15
Rumors of change	3142	1.00	0.84–1.18
Organizational procedural injustice	2884	1.08	0.89–1.30
Commitment to organization	3068	0.93	0.72–1.19
Positive challenge	2954	0.70	0.51–0.96**
Human resource primacy	2986	0.77	0.58–1.01
Social climate	3105	0.74	0.55–1.01
Experienced bullying^b^	2944	2.17	0.98–4.81
Observed bullying^b^	3126	1.28	0.74–2.23

a Separate regressions were run for each factor and subjects reporting mental distress at baseline were excluded. Age, sex, skill level, and mental distress at T1 as a continuous variable were included in all regressions.

b Response categories were yes/no.

**p<.01.

***p<.0005, which was the bonferroni-adjusted threshold of statistical significance.

#### Average exposure as predictor

Univariable analyses revealed that all factors except *quantitiative demands*, *decision demands*, and *predictability during the next two years* were related to mental distress (p<.01; analyses not shown). In addition, the category “observed T1, not T2” of the factor *observed bullying* was not statistically significant. After Bonferroni-correction all factors except *control over work intensity* and “bullied T1, not T2” of *experienced bullying* remained statistically significant. All random intercept models showed better fit to the data compared to models also including random slopes. The differences in BIC values were >10 for all models except for *decision demands* (difference of 7.6) (not shown).

Multivariable regressions showed that statistical significance was maintained for all work factors except *control over work intensity* (Table 3). *Role conflict, rumors of change, procedural injustice, observed bullying* (“observed T2 only”), and *experienced bullying* (“bullied T2 only” and “bullied both T1 and T2”) increased the odds of mental distress. *Decision control, role clarity, support from immediate superior, empowering leadership, fair leadership, predictability during the next month, commitment to organization, positive challenge, human resource primacy*, and *social climate* were associated with decreased odds. All factors except *procedural injustice* and “bullied both T1 and T2” of *experienced bullying* remained statistically significant after Bonferroni-correction. The BIC values were smaller for random intercept models with differences of >10 for all models except for *decision demands* (difference of 9.1) (not shown). Hence, adding random slopes to the models did not improve the fit.

**Table 3:** Multivariable random intercept logistic regression models with psychological and social work factors averaged across time ([T1+T2]/2) as predictors of mental distress above the cut-off for "caseness" at T2^a^.

Exposure	N	OR	99% CI
Quantitative demands	3008	1.21	0.87–1.68
Decision demands	2910	0.78	0.52–1.16
Decision control	2864	0.58	0.39–0.86***
Control over work intensity	3086	0.85	0.67–1.07
Role conflict	3106	2.08	1.45–3.00***
Role clarity	3121	0.57	0.41–0.78***
Support from immediate superior	3049	0.56	0.43–0.72***
Empowering leadership	3111	0.64	0.51–0.81***
Fair leadership	3037	0.52	0.40–0.68***
Predictability during the next month	3115	0.65	0.47–0.90***
Predictability during the next two years	2680	0.85	0.66–1.09
Rumors of change	3111	1.32	1.06–1.63***
Organizational procedural injustice	2663	1.30	1.01–1.66**
Commitment to organization	3045	0.65	0.50–0.86***
Positive challenge	2797	0.60	0.41–0.86***
Human resource primacy	2861	0.53	0.38–0.74***
Social climate	3050	0.43	0.31–0.61***
Experienced bullying^b^	2780		
Not bullied T1 or T2		ref	
Bullied T1 only		2.14	0.77–5.95
Bullied T2 only		3.37	1.45–7.82***
Bullied both T1 and T2		4.44	1.15–17.07**
Observed byllying^b^	3073		
Not observed T1 or T2		ref	
Observed T1 only		1.05	0.50–2.19
Observed T2 only		2.41	1.28–4.52***
Observed both T1 and T2		2.24	0.98–5.13

a Separate regressions were run for each factor and subjects reporting mental distress at baseline were excluded. Age, sex, skill level, and mental distress at T1 as a continuous variable were included in all regressions.

b Response categories were yes/no.

**p<.01.

***p<.0005, which was the bonferroni-adjusted threshold of statistical significance.

## Discussion

To our knowledge, this is the first study that has investigated such a *broad set* of *specific* psychological and social work factors as predictors of potentially clinically relevant mental distress (anxiety and depression) with a prospective, full-panel design. In addition to shedding light on processes driving the relationship of work with mental distress, this knowledge should offer a practical starting point for efforts to improve working conditions and health. Fourteen of 19 factors showed some prospective association with incidence of mental distress (Table 2 and 3). *Quantitative demands*, *decision demands*, *control over work intensity*, *predictability during the next two years*, and *observed bullying* were not statistically significant predictors. *Role conflict, support from immediate superior, fair leadership*, and *positive challenge* were *consistent* predictors. We have labelled as consistent those factors that were statistically significant in both analyses of baseline exposure *and* average exposure over time.

Considering the wide range of work factors included in the present study, an elaborate discussion of each factor is beyond the current scope. Therefore, we focus on previously less investigated factors. Also, important differences from previous findings will be addressed.

The most consistent *risk factor* in the present study was *role conflict* (e.g. “receive incompatible requests from two or more persons”). The item “conflicting demands” of the *job demands* factor of Karasek's JCQ [10] overlaps with “role conflict” as measured by QPS_Nordic_ in the current study. One prospective study reported that role conflict (QPS_Nordic_) predicted level of mental distress (HSCL-10) among nurses' aides [47]. In a 3-year follow-up study of the general working population in Norway, role conflict (QPS_Nordic_) predicted subsequent mental distress (measured by two single items) [48]. Furthermore, role conflict (QPS_Nordic_) was among the strongest predictors of neck pain in a recent prospective study including many of the same work factors as the present study [13]. Hence, role conflict seems to represent a substantial health risk.

Systematic reviews have concluded that *psychological job demands* (most often assessed by Karasek's JCQ) predict depression and anxiety [3], [4], [6], [7], [8], [9]. Q*uantitative demands* (QPS_Nordic_) reflecting work amount which is one aspect of “job demands” of JCQ was not a risk factor in the present study. Hence, the present results on quantitative demands seem inconsistent with previous findings on “job demands”. However, “job demands” from Karasek's JCQ includes the item “conflicting demands” which seems to overlap with the current measure of role conflict, which was a consistent risk factor. Thus, the components included in the job demands concept of JCQ [10] may be differentially related to mental distress with role conflict as the most significant component.

*Observed bullying* increased the risk of mental distress in the present study. However, a long-term effect was not discovered as the only statistically significant result in prospective analyses (with average exposure) was for the category “observed at T2 only”. One previous prospective study has reported that observed bullying was a risk factor for depressive symptoms [49]. However, since observers of bullying may be victims of bullying as well, this relationship has been suggested to be confounded [50].

The present results showed *predictability during the next month* and *rumors of change* to be associated with mental distress. In previous studies *job insecurity* (i.e. threat of job loss) has been reported to predict common mental disorders [9]. Predictablity and rumors of change differ from job insecurity. The “predictability during the next month” scale assesses if employees know which tasks and coworkers to expect in one month. To our knowledge, no previous prospective studies have investigated predictability in relation to mental distress. A prospective study of predictors of long-term sickness absence due to psychiatric illness did not find an effect of job insecurity. One of the items related to job insecurity was the question of rumors of change (QPS_Nordic_) [51]. An independent effect of rumors of change on depressive symptoms was reported in a prospective study of industry workers [49].

High scores on the *social climate* scale were related to a reduced risk of mental distress. Three aspects of social climate (encouraging and supportive; distrustful and suspicious; relaxed and comfortable) were assessed. In a study of nurses' aides the effect of social climate (QPS_Nordic_) on distress did not persist when adjusting for demographic factors and other work exposures [47]. One reason for this could be that some of the included work factors mediated the effect. Knowledge regarding how different work factors may interact in complex mechanisms influencing health is limited. *Human resource primacy* (e.g. “are workers well taken care of in your organisation”) was found to reduce the risk of mental distress in analyses with average exposure. As far as we know, human resource primacy has not been investigated prospectively in relation to mental distress.

*Empowering leadership* contributed to a reduced risk of mental distress. We have not found prospective studies investigating empowering leadership in relation to mental distress. Transformational leadership (i.e. leaders that inspire to making own decisions and facilitate the development of individuals by providing personal attention, support, and a visionary and creative leadership style) may protect against depression [52]. However, depression at baseline was not taken into account in the study by Munir, Nielsen, and Carneiro [52]. An index comprising empowering-, fair-, and supportive leadership (QPS_Nordic_) has been found to predict general health [53]. Empowering leadership (QPS_Nordic_) has also been found to be a strong protective factor for neck- [13] and back pain [14]. Hence, a leader that encourages employees to participate in important decisions, to speak up when having different opinions, and helps employees to develop their skills [37] seems to be an important protective factor across different health outcomes.

In agreement with the present study, previous studies have found *decision control*, *positive challenge* (resembles “skill discretion” of Karasek's JCQ) [3], [4], [6], [7], [8], [9], *social support* [3], [4], [6], [7], [9], *organisational justice* [3], [7], [5], and *experienced bullying* [3], [40] to be explanatory factors for mental health problems.

Work factors may contribute to mental health through several pathways. Mastery and self-esteem have been suggested as mediators in a cross-sectional study [54]. Feelings of mastery at work may be particularly vulnerable to task level factors (control, role expectancies, and predictability during the next month) [37]. For instance, low control or high levels of role conflict could interfere with the accomplishment of work tasks and thereby reduce the experience of mastery. Social- and organizational level factors (social interaction, leadership, and organizational climate) [37] may be particularly important for self-esteem. Receiving support and experiencing a positive social climate could improve the individual's evaluation of self worth. Reduced self-esteem may result from the experience of being bullied (e.g. [55]).

Overactivation and dysregulation of the hypothalamic-pituitary-adrenocortical (HPA) axis may be produced by work factors (see [56]). Elevated arousal could result from worrying about time pressure or tasks being too demanding or when expectations are in conflict. Worrying or ruminating about time pressure or tasks being too demanding or when expectations are in conflict could affect sleep and restitution [57] and influence relationships with significant others. Almost all neurohumoral systems are influenced by arousal and dominance-subordinance interactions.

The current study investigated exposures separately to identify relevant risk factors. However, these factors may interact in complex causal processes influencing mental distress. For instance, non-supportive leadership [58], role conflict [58], [59], and role ambiguity [59] have been related to increased prevalence of workplace bullying. In the present study, role conflict, supportive leadership, and bullying were predictors of mental distress. Possible associations between work factors could influence the results of intervention efforts. Interventions pertaining to one particular exposure may have favourable effects on other factors. For example, reducing the level of role conflict may also reduce the prevalence of bullying.

A larger number of associations were significant in the average-exposure design compared to in the baseline-exposure design. This may indicate that enduring exposure is more likely to produce mental distress. Another possible explanation is that effects of many work exposures are short-term [60]. Hence, some exposures measured at baseline may not produce effects that endure until follow-up. Cross-sectional analyses of T1 and T2 data (not shown) showed that all exposure factors except *decision demands* at T2 were associated with mental distress, supporting the interpretation that exposure level at T2 contributes to distress at T2. Moreover, the approach of averaging over time points should yield more “reliable” estimates of the overall working conditions over the time period in question. Hence, including more than one single assessment of exposure seems important. *Empowering leadership* was one of the factors that were statistically significant only in analyses with average exposure. Possibly, this indicates that the influence of empowering leadership was mainly short-term *or* produced by enduring exposure rather than resulting from baseline exposure across a time period of two years. However, since only two waves of data were available, average exposure measures included exposure at T2, which implied a cross-sectional element in the analyses that must be taken into account when interpreting results. This element curtails the extent to which *causal* inference can be made. Nevertheless, for factors that are valid predictors at baseline, a stronger average-exposure effect may signify the significance of taking into account exposure levels over extended periods of time.

### Methodological considerations

The baseline response rate for individuals invited to the first survey was 51.6%. The attrition rate from baseline to follow-up was 32.6%. Non-response and attrition may affect the *external validity* if those not participating differ from those who do. Differences were discovered in age (non-response and attrition), sex, skill-level, and work factors (attrition) (see Table 1 and “Results”). However, although the current study included a diverse sample the *exact* population to which generalisation was valid cannot be determined *a priori* since it is unknown whether or not the invited employees were representative of the Norwegian (or international) working population. Hence, this selection has a limited impact on the external validity of the study. *Internal validity* may be threatened if self-selection is related to both exposures and outcome [61]. Attrition analyses showed that some of the exposures predicted responding at follow-up. However, mental distress at baseline was not related to response (Table 1).

Self-report measures of predictors and outcome may be affected by the same reporting biases (e.g. due to negative affectivity). Correlated measurement errors may inflate associations between work factors and mental distress (*common method bias*, CMB) [62]. Longitudinal studies are less prone to CMB due to temporal separation of measurements (e.g. situational factors inducing negative or positive states are not likely to occur at both measurement occasions). Also, the way QPS_Nordic_ is constructed should attenuate reporting biases [37], [62]: terms with negative/positive connotations (e.g. “satisfied with”) were avoided in response scales and people were asked how often a situation occurs (i.e. frequency), verbal labels were used for all response categories, and some items were reversed. In addition, exposure- and effect measurements were placed in different sections of the questionnaire and rated on different scales, and respondents were assured anonymity. Excluding baseline “cases” and adjusting for mental distress as a continuous variable in the prospective analyses should attenuate CMB [62]. Furthermore, associations between psychological and social work factors and mental health problems have also been found in studies using externally assessed exposures (i.e. by observation and interview) [63], [64], diagnostic interviews as outcome (see systematic reviews: [3], [5], [6], [8], [9], and studies taking into account the potential confounding effect of personality traits [65], [66]. Hence, one cannot conclude that observed relations are due to common method bias. However, an influence on the effect estimates can not be ruled out.

Exclusion of baseline “cases” and adjustment for baseline distress protect against type I errors. However, type II errors may occur if mental distress reported at baseline was influenced by previous or baseline exposure. Mental distress at baseline was the strongest predictor of mental distress at follow-up. Furthermore, statistical power may be reduced by information loss due to dichotomization. However, dichotomization was necessary as the outcome of interest was clinically relevant mental distress.

The present study demonstrated that a large number of psychological and social work factors contributed to mental distress. *Role conflict* was the most consistent risk factor and *support from immediate superior, fair leadership*, and *positive challenge* were the most consistent protective factors. Thus, it is important for future studies to broaden the scope and include factors beyond the demand-control model and the effort-reward imbalance model. The current results of a variety of *specific* factors at both the task-, individual-, and social- and organizational level provide knowledge for designing workplace interventions aiming to reduce mental distress among employees. Knowledge of specific work factors that contribute to health provides a better basis for practical efforts to improve occupational health by: i) specifying factors to measure in surveys/assessments, and ii) focusing targets of interventions or changes.
